# Homo- and Hetero-Oligonuclear Complexes of Platinum Group Metals (PGM) Coordinated by Imine Schiff Base Ligands

**DOI:** 10.3390/ijms21103493

**Published:** 2020-05-15

**Authors:** Barbara Miroslaw

**Affiliations:** Department of General and Coordination Chemistry and Crystallography, Institute of Chemical Sciences, Faculty of Chemistry, Maria Curie-Sklodowska University in Lublin, Pl. Marii Curie-Sklodowskiej 3, 20-031 Lublin, Poland; barbara.miroslaw@poczta.umcs.lublin.pl; Tel.: +48-815-375-582

**Keywords:** oligonuclear complexes, platinum group metals, PGM, Schiff base, imine ligand, coordination compound

## Abstract

Chemistry of Schiff base (SB) ligands began in 1864 due to the discovery made by Hugo Schiff (Schiff, H., *Justus Liebigs Ann. der Chemie* 1864, *131* (1), 118–119). However, there is still a vivid interest in coordination compounds based on imine ligands. The aim of this paper is to review the most recent concepts on construction of homo- and hetero-oligonuclear Schiff base coordination compounds narrowed down to the less frequently considered complexes of platinum group metals (PGM). The combination of SB and PGM in oligonuclear entities has several advantages over mononuclear or polynuclear species. Such complexes usually exhibit better electroluminescent, magnetic and/or catalytic properties than mononuclear ones due to intermetallic interactions and frequently have better solubility than polymers. Various construction strategies of oligodentate imine ligands for coordination of PGM are surveyed including simple imine ligands, non-innocent 1,2-diimines, chelating imine systems with additional N/O/S atoms, classic N_2_O_2_-compartmental Schiff bases and their modifications resulting in acyclic fused ligands, macrocycles such as calixsalens, metallohelical structures, nano-sized molecular wheels and hybrid materials incorporating mesoionic species. Co-crystallization and formation of metallophilic interactions to extend the mononuclear entities up to oligonuclear coordination species are also discussed.

## 1. Introduction

The present paper focuses on homo- and hetero-oligonuclear Schiff base (SB) complexes of platinum group metals (PGM), which are rarely considered in numerous comprehensive reviews despite of their great importance.

**Figure 1:**
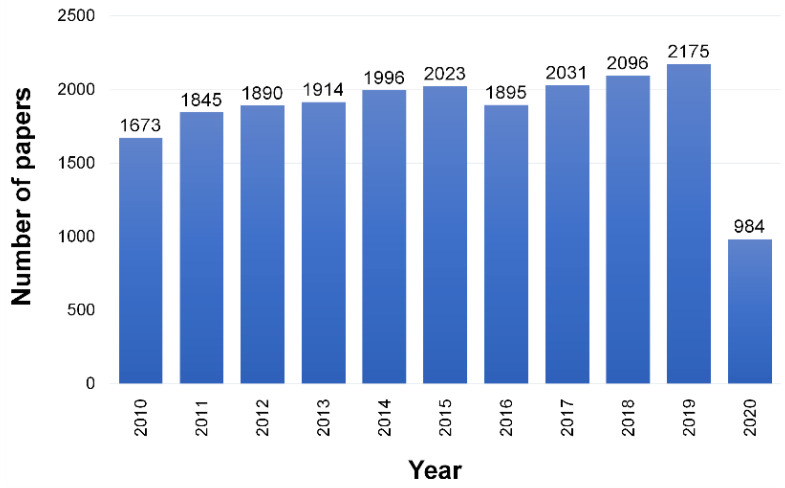
Number of papers published per year with the concept “Schiff base” in the title, abstract or keyword section according to Scopus document search in the period of the last 10 years (data retrieved on 08 May 2020).

The reaction between carbonyl compound and primary amine discovered by Hugo Schiff in 1864 [1] gave basis for further research in various scientific fields and resulted in thousands of papers being submitted by scientists all over the world to a diverse spectrum of scientific magazines. Over the past 10 years, approximately 2000 new papers were published annually according to Scopus document search (Figure 1). The reports cover synthesis of new organic and coordination compounds, their luminescent, magnetic, catalytic or bioactive properties up to advanced applications in many industries such as dye-sensitized solar cells. Although the topic seems to be quite old and exploited, several scientific groups worldwide are still studying the great potential of this group of compounds finding new applications for the imine based ligands and their complexes. The azomethine molecular fragment is used as a structural element for the synthesis of polydentate ligands of various structures up to mechanically interlocked molecular architectures such as metallacycles, cages or knots [2,3,4].

Worth recommending are excellent reviews on life and work of Hugo Schiff [5,6] who was not only a formidable scientist but also an interesting personality actively engaged in civic and political life. For a better insight into this section of chemistry, it is worth to refer to comprehensive reviews on chemistry and general information on imines, which were widely summarized by Layer [7] and more recently by other scientific groups [8,9,10,11,12,13]. Zoubi et al. reported the recent progress in thermal analysis of Schiff base [14].

### 1.1. Synthesis Methods of Schiff Base Ligands and Their Complexes

The classic synthesis method of Schiff bases (SB) is a condensation of carbonyl compound with primary amine carried out in solution, where solvents are usually organic compounds forming azeotropic mixtures. This method allows one to construct ligands ranging from small molecules up to macrocyclic species and covalent organic-frameworks based on SB chemistry [9,15,16,17,18,19]. The mechanism of SB formation and hydrolysis was discussed in details by Cordes and Jencks [20]. Water is regarded as a disturbing factor inhibiting the progress of the chemical reaction, which can be eliminated by molecular sieves [21], or dehydrating agents acting as catalysts such as phosphorous(V) oxide supported on silica [22], magnesium perchlorate [23], titanium(IV) chloride [24], aluminum chloride [25] and hydrobromic acid [26]. Apart from conventional methods, there are also more sophisticated ones, including green chemistry methods with synthesis performed in water suspension medium [27] or under solvent-free conditions supported by using the microwave irradiation [28] and montmorillonite K 10 clay [29] or tungsten(VI) chloride as [30] catalyst, or on alumina surface [31]. Rao et al. reported an eco-friendly synthesis method in aqueous medium [32]. Among reports on synthesis of SB worth mentioning are the methods performed in ionic liquids [33], techniques with the use of ordered mesoporous materials [34], solvent free synthesis reaction performed under infrared [35], ultrasound [36] and microwave irradiation [37]. Direct synthesis mechanochemical methods are also applied to perform solid-solid synthesis [38,39,40] confirming the high reactivity of this group of compounds.

The synthesis of coordination compounds of SBs may be performed in a stepwise manner, i.e., first synthesis of the SB ligand and subsequently the synthesis of the complex [41,42]. The modular “chemistry on complex” approach can be applied with the use of metal cross-coupling reactions [43]. However, frequently the obtaining of ligands and complexes may be conducted in situ by one-pot reaction with simultaneous addition of SB substrates and metal salts resulting in simple self-assembling of N_2_O_2_ compartmental SB coordination units [44] up to macrocyclic calixsalens [45]. The metallic species may play a catalytic and template role in such syntheses. The details of many interesting and efficient synthesis methods for SB complexes can be found in the cited research reports.

### 1.2. Importance and Application of Schiff base Ligands and Their Complexes

SBs play a crucial role in catalytic mechanisms of biologically important proteins [46]. There are also reports on Schiff base acting as an intermediate in the Maillard reaction resulting in the formation of Amadori or Heyns products such as acrylamide from sugars and amino acids during high-temperature cooking processes (i.e., frying, roasting or baking). This type of rearrangement reactions may be responsible for the pathological effects of diabetes, Alzheimer’s disease and ageing processes in general [47,48,49,50].

Imine compounds and their metal complexes exhibit a broad range of biological activities such as anti-inflammatory, antiviral, antifungal, antibacterial, antimalarial, antiproliferative, antipyretic properties, etc. The excellent reviews on biological activities of SBs can be found in the following references [51,52,53,54,55,56,57,58]. SB complexes can be used as urease inhibitors [59]. Sztanke et al. summarized the antiprolife activity of azomethine derivatives [60]. The SBs are recognized as a versatile pharmacophore [57]. Polynuclear salen-mesoionic carbene hybrid complexes have application as biomedical hydrogels [61]. The complexes of Schiff bases with lanthanides find many applications for example as a contrast media for magnetic resonance imaging or in the therapy of neoplastic diseases [62]. Majid et al. reviewed the capability of SB complexes to enhance the transport across the biomembrane [63].

Many compelling reviews report on versatile applications of SBs and their complexes [13,64]. Catalysis is another field of application of SB ligand and their complexes. Many SB complexes show excellent catalytic activity and can be used in organic synthesis [12,54,65,66,67,68,69,70]. SBs can be applied for solvent extraction of metal ions [71,72,73]. Schiff Bases and their transition metal complexes are applied as an efficient optical chemical [74,75,76] or electrochemical sensors for detection of analytes in forensic, pharmaceutical and environmental samples [74,77,78,79,80]. Oligonuclear PGM complexes are assessed as prospective phosphorescent emitters in organic light-emitting diodes (OLEDs) [81]. The SB compounds are also used in nanotechnology as coatings for chemical sensing [82] and as supramolecular sensing materials for preparation of potentiometric membrane sensors [83]. The complexes of SBs are applied for production of dye-sensitized solar cells [84,85,86,87]. The utilization of SB complexes including PGM oligomers as energy storage materials for batteries and supercapacitors was summarized by Zhang [88].

### 1.3. Schiff base Ligands in Coordination Chemistry

Due to excellent ligating properties and relatively facile synthesis methods the imine based ligands are widely used in transition metal coordination chemistry. A collection of interesting papers on SB metal complexes was published recently in special issues in *Inorganics* edited by Professor Dr. Santo Di Bella and in *Molecules* edited by Professor Dr. Antonella Dalla Cort [89,90]. Jain and Jain published a comprehensive review in 2005 with nearly two thousand of references exclusively on binuclear palladium(II) and platinum(II) complexes [91]. Golbedaghi and Fausto reviewed the cocrystal formation of SBs [92] together with their application in pharmaceutical, paper, textile, photographic and electronic industries. Beck and Sünkel surveyed the most recent achievements in coordination compounds of bidentate indigo related ligands [93]. Multi-metallic salen complexes were described by Mondal and Chattopadhyay [94] and penta-, hexa- and heptadentate SBs by Liu and Hamon [12,13]. Karmakar and Chattopadhyay presented the stereochemistry of tetradentate N_2_O_2_ donor Schiff base ligands in octahedral complexes [95]. However, the authors focused on trivalent 3d metals. The structural aspects of SB and their complexes can be found in excellent reviews for example on multi-metallic salen [94], arene based complexes [96] and salicylaldehyde [97] complexes, or on actinoid oligonuclear complexes of SB ligands [98]. Liu and Hamon summarized the penta-, hexa- and heptadentate SB ligands and their complexes [13]. The SB ligands were also studied in the macrocyclic forms of multidentate aza, oxoaza or thiaaza heterocyclic compounds [16] or oligo- and oligometalloporphyrins [99]. Additionally, the tautomerism of SBs and possibility of hydrogen bond or stacking interaction formation was studied intensively together with layer-by-layer (LbL) assembly for many applications [100,101,102,103,104,105,106,107,108].

There were published many reviews on Schiff base ligands and their coordination compounds. However, oligonuclear SB coordination compounds with PGMs are much less often taken into consideration and frequently omitted in discussions. The current manuscript focuses on this particular interesting combination of imine ligands and platinum group metals (i.e., Ru, Rh, Pd, Os, Ir and Pt) collecting in one paper the most recent and important advances in this field.

Due to the extraordinary catalytic, luminescent and magnetic properties of this unique group of compounds the perspective application of oligonuclear SB-PGM materials in modern technology is versatile.

This review focuses on the significant reports on obtaining oligonuclear SB-PGM materials published over the past ten years, i.e., 2010-2020. However, some earlier studies (down to 1980) were also included because of their importance. This review may offer a support in the rational design and construction of new oligonuclear platinum group metal coordination compounds incorporating Schiff base building blocks for high tech applications in material science as energy or gas storage materials, in catalysis, electrochemical or luminescent sensors and/or for biomedical usage.

## 2. Results and Discussion

### 2.1. Strategies on Construction of Oligonulear Schiff Base Complexes of Platinum Group Metals

The present review is divided into eight sections related to various structural approaches to obtain homo- and hetero-oligonuclear coordination PGM compounds incorporating imine ligating sites. In the first section the use of simple imine as a monodentate or bridging ligand is discussed. Then following section surveys the unusual behavior of non-innocent 1,2-diimine related ligands, which may ligate both in a *σ*- and *π*-fashion. Next, the examples are given on combining the imine group with additional ligating N/O/S atoms affording coordination chelating pockets. The usage of widely studied N_2_O_2_ Schiff base compartmental ligands is summarized in the next section. Apart from classic bi- or trinuclear PGM systems based on N_2_O_2_ compartment SB ligands, the concept of utilization this system as building blocks for the construction of oligodentate species in the form of expanded voluminous molecules, macrocycles, tweezers or knots is presented.

The last sections show the approach to application of auxiliary linking ligands, cocrystals formation and utilization of metallophilic interactions to increase the nuclearity of Schiff base related PGM coordination compounds.

The extensive topic of ligands with imine group built in cyclic species such as azoles, purine bases, porphyrins, etc. was excluded from this review due to vast literature in this field.

**Scheme 1:**
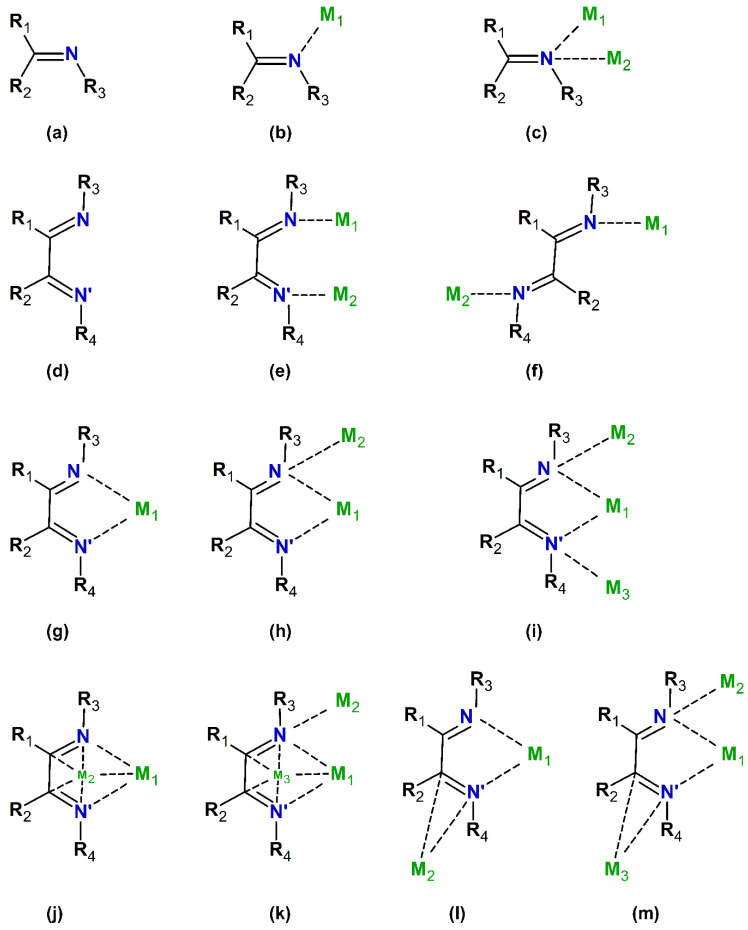
Coordination modes of imine and 1,2-diimine ligands with denticity of ligands described with kappa notation (*κ*) and where necessary hapticity given in eta notation (*η*): (**a**) Imine ligand; (**b**) monodentate 2e *κ*^1^ mode; (**c**) monoatomic bridging 2e *μ*_2_-*κ*^2^ mode; (**d**) 1,2-diimine ligands; (**e**) bidentate bridging 4e *μ*_2_-*κ*^1^:*κ*^1^ mode in *s-cis* 1,2-diimine; (**f**) bidentate bridging 4e *μ*_2_-*κ*^1^:*κ*^1^ mode in *s-trans* 1,2-diimine; (**g**) bidentate chelating 4e *κ*^2^-N,N’ mode; (**h**) tridentate chelating bridging 4e *μ*_2_-*κ*^2^:*κ*^1^ mode; (**i**) tetradentate chelating bridging 4e *μ*_3_-*κ*^1^:*κ*^2^:*κ*^1^ mode; (**j**) 8e *μ*_2_-*κ*^2^-N,N’:*η*^2^-C=N:*η*^2^-C’=N’ mode; (**k**) 8e *μ*_3_-*κ*^1^-N:*κ*^2^-N,N’:*η*^2^-C=N:*η*^2^-C’=N’ mode; (**l**) 6e *μ*_2_-*κ*^2^-N,N’:*η*^2^-C’=N’ mode and (**m**) 6e *μ*_3_-*κ*^1^-N:*κ*^2^-N,N’:*κ*^1^-N’:*η*^2^-C’=N’ mode.

**Scheme 2:**
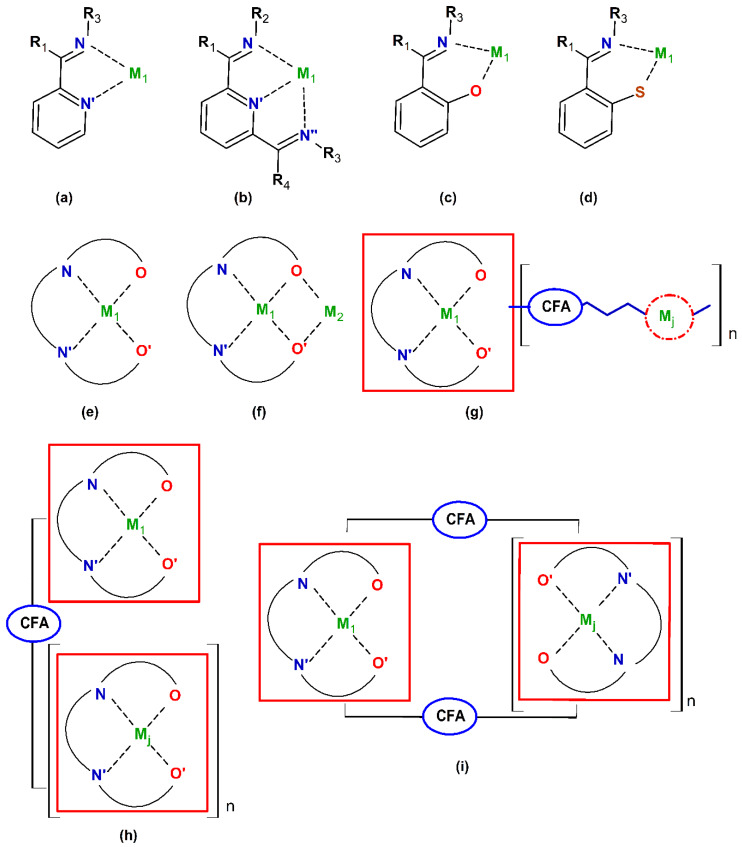
(**a**–**d**) Coordination chelating pockets made of imine group and additional ligating heteroatom N/O/S; (**e**) scheme of binuclear and (**f**) trinuclear complex based on N_2_O_2_ compartmental Schiff base ligand; (**g**) hybrid material made of SB fused by covalently fusing agent (CFA) with additional ligating moieties, *n* ≥ 1, *j* ≥ 1; (**h**) acyclic and (**i**) cyclic fused SB units resulting in oligodentate ligands.

The summarized coordination modes of imine, non-innocent 1,2-diimine, coordination chelating pockets based on azomethine and additional N/O/S ligating atoms and N_2_O_2_ Schiff base compartmental ligands are presented in Scheme 1 and Scheme 2.

### 2.2. Imine as a Monodentate and Bridging Ligand

**Figure 2:**
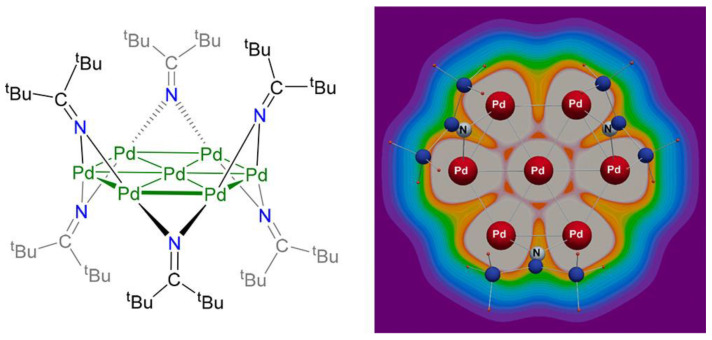
Hexagonal Pd_7_ nanosheet stabilized by ketimine ligand [109]. Reprinted with permission from A. W. Cook, P. Hrobárik, P. L. Damon, G. Wu, T. W. Hayton, *Inorg. Chem.* 2020, 59, 2, 1471–1480. Copyright (2020) American Chemical Society (CSD Refcode PIDCID).

The simple imine C=N molecular fragment is a powerful key to coordination chemistry (Scheme 1a). The most important advantages of azomethine group in coordination chemistry are (1) simple and efficient synthesis enabling linking of various larger molecular fragments and (2) high affinity to form coordination bonds with all metallic elements. The imine N atom can act as a monodentate or bridging ligand (Scheme 1b,c) resulting in marvelous structures such as reported by Cook at al. [109]. A unique hexagonal Pd_7_ nanosheet (Figure 2) was synthesized via the reaction of PdCl_2_(PhCN)_2_ and Li(N=C*^t^*Bu_2_) in THF at low temperature of −25 °C. The structure creates an opportunity to study the exceptional coordination environment of the central palladium(0) atom. This report shows that ketimines may be effective agents at stabilizing nanoclusters of low-valent transition metals. This structure represents an “Atomically Precise NanoCluster” (APNC), which usually is related to Au, Ag and Cu nanostructures applied for high tech solutions in catalysis, imaging and quantum computing [110,111,112].

**Figure 3:**
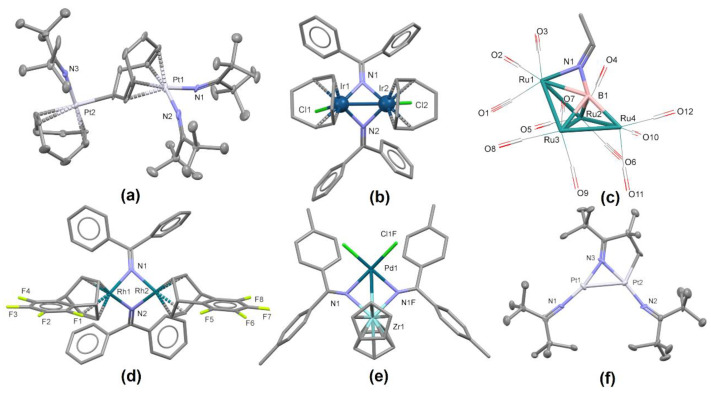
(**a**) Binuclear complex of Pt(II) with monodentate *κ*^1^-N ketimine ligands and activated 1,5-cyclooctadiene fragment acting as an intermetallic bridge [113] (CSD Refcode MOYJES); (**b**) binuclear complex of Ir(II) with ketimine as an intermetallic bridge and 1,5-cyclooctadiene ligands located on the outside of the coordination unit [116] (CSD Refcode KABLOP); (**c**) bridging *μ*_2_-*κ*^2^ ethanimine ligand in metallaborane tetra-ruthenium cluster with boron–nitrogen coupling [117] (CSD Refcode PORSUK); (**d**) binuclear complex of Rh(II) with ketimine bridging ligand [118], shown only one symmetrically independent molecule (CSD Refcode LIFMIW); (**e**) heterobimetallic Zr/Pd complex with ketimine linkage [119] (CSD Refcode WAGHOC) and (**f**) binuclear complex of Pt(II) stabilized by ketimine ligand with very short Pt–Pt distance and activated C–H bond [113] (CSD Refcode MOYHUG). Solvent molecules and hydrogen atoms were omitted for clarity.

A simple ketimine ligand may yield complexes coordinated in the *κ*^1^-N monodentate mode. An example is a binuclear complex of Pt with additional activated 1,5-cyclooctadiene fragment acting as a bridge between two metal centers (Figure 3a; MOYJES) [113]. Secondary ketimine ligands were also utilized as monodentate ligands supplementing the coordination sphere in complexes of Rh [114] and Pd [115].

However, the ketimine fragment may also play a role as a bridging ligand itself. Werner et al. reported a reversed structure with ketimine as a bridge and 1,5-cyclooctadiene located on the outside of the coordination unit (Figure 3b; KABLOP) [116].

A simple bridging *μ*-*κ*^2^ ethanimine ligand may also play an auxiliary role as in the case of metallaborane cluster found in the complex of (*μ*_4_-ethyliminohydridoboron-H,B,B,B,B,N)-(*μ*_2_-hydrido)-dodecacarbonyl-tetra-ruthenium (Figure 3c; PORSUK), which was studied in photolysis. This is also an example of coupling between N and B atoms [117].

Ketimine bridging ligands were also used to synthesize complexes of other platinum group metals such as Rh, Ir [118] or Pd [120] (Figure 3d; LIFMIW). Such compounds may be applied for imine-directed aromatic C–H bond activation [121].

Kuwabara et al. used the ability of ketimine ligand to form intermetallic bridges for synthesis of very interesting coordination architecture of mixed Zr/Pd heterobimetallic complex (Figure 3e; WAGHOC) [119], where the coordination sphere was supplemented by *η*^5^-cyclopentadienyl ligands and chloride anions.

The application of simple ketimine ligand resulted also in the first report on linear Pt(II) complex of Pt(N=C^t^Bu_2_)_2_ and a binuclear byproduct [(^t^Bu_2_C=N)Pt(*μ*-N,C–N=C(^t^Bu)C(Me)_2_CH_2_)Pt(N=C^t^Bu_2_)] with metallophilic interaction giving very short Pt–Pt distance (2.5951(6) Å) and activated one of the *t*-butyl C–H bonds (Figure 3f; MOYHUG) [113]. This structure breaks the well-established knowledge on planar tetragonal coordination requirements for the Pt(II) metal center. Additionally, this binuclear construction shows why the PGM complexes are so special in comparison to other transition metals. They deliver additional tools for crystal engineering in the form of metallophilic interactions and bond formation between metal and C atoms.

### 2.3. Non-innocent 1,2-diimine Ligands Binding in σ- and π-fashion

**Figure 4:**
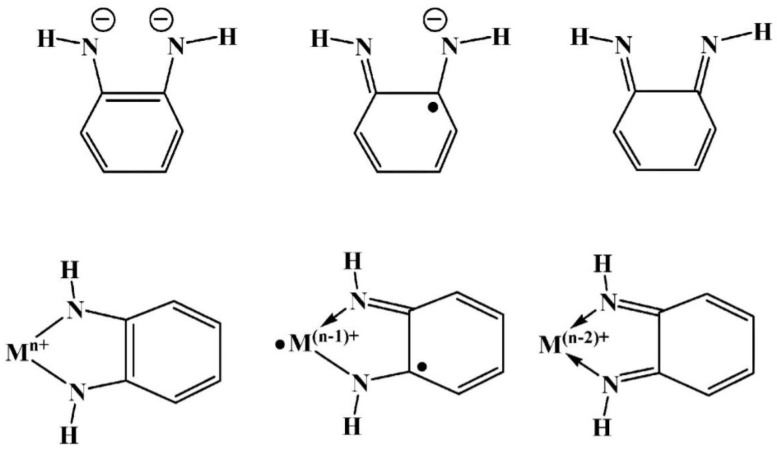
The behavior of non-innocent ligand upon complexation [122]. Reprinted with permission from Anillo, A.; Díaz, M. R.; García-Granda, S.; Obeso-Rosete, R.; Galindo, A.; Ienco, A.; Mealli, C. Structural and electronic rearrangements upon the oxidation of binuclear (Ru_2_) and trinuclear (MoRu_2_) complexes with bridging *o*-phenylenediamido ligands. Organometallics 2004, 23 (3), 471–481. Copyright (2004) American Chemical Society.

1,2-Diketimines (Scheme 1d) belong to the group of “non-innocent ligands”, which means that in metal complexes their oxidation state is not clear. The non-innocent ligands, when combined with redox–active transition metal ions, can result in redox-tautomeric complexes (Figure 4) [122]. The characteristic feature for these complexes is the intramolecular electron transfer between the coordinated ligand and the metal centers as well as intermetallic electronic interactions [123,124,125,126]. Additionally, such complexes may exhibit low-energy electronic transitions (NIR electrochromism) that can be used in optical materials for example as variable optical attenuator [127,128]. Kaim published an excellent review on chelate rings formed by non-innocent ligands [129].

The application of 1,2-diketimines for synthesis of PGM oligonuclear complexes may result in unusual structures due to the ability of this ligand to bind both via *σ*, as well as, *π*-bonding (Scheme 1e–m). 1,2-Diimine is a versatile building element used for making bridges between coordination centers. In acyclic entities this type of ligand may have *s-cis* or *s-trans* arrangement, which may lead to a different ligating function.

**Figure 5:**
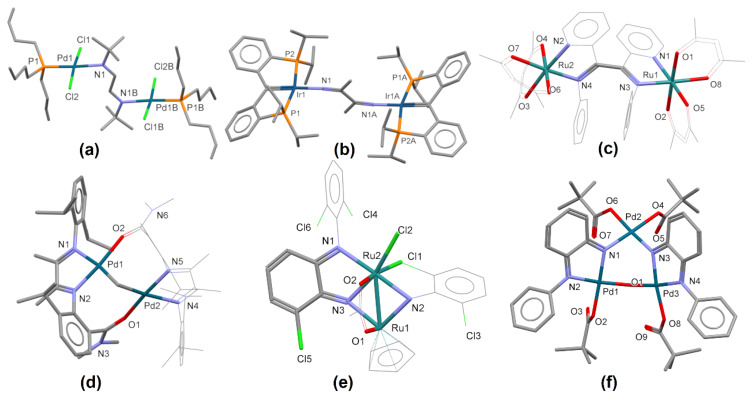
(**a**) A mixed homo- and heterobimetallic palladium-platinum complex with the bridging 4e donor *σ*,*σ*-N,N’ coordination mode [132] (CSD Refcode FACKAV); (**b**) butane-2,3-diiminato ligand synthesized in situ via reductive coupling of acetonitrile at the iridium metallic center [133] (CSD Refcode SEYKOY); (**c**) unusual synclinal conformation of the bridging 1,2-diimine ligand in the meso-(*μ*-N,N’-diphenyl-1,2-di(pyridin-2-yl)ethane-1,2-diimine)-tetrakis(acetylacetonato)-bi-ruthenium complex [134] (CSD Refcode LOMNIN); (**d**) bidentate chelating 4e *κ*^2^-N,N’ binding mode of *s-cis* diimine moiety in the dipalladium complex [135] (CSD Refcode ADOGEI); (**e**) tridentate chelating bridging *μ*_2_-*κ*^2^:*κ*^1^ mode of the coordination of *s-cis* 1,2-diiminebenzene related ligands [136] (CSD Refcode KIGFUD); (**f**) chelate and bridging ligation by 1,2-diiminebenzene based ligands in potassium bis(*μ*-N-phenyl-o-phenylenediimiine)-(*μ*-hydroxo)-tetrakis(pivalato)-tri-palladium(II)xylene solvate dihydrate [137] (CSD Refcode MICDAD). Hydrogen atoms, solvent and counterion molecules were omitted for clarity.

A simple bridging 4e donor *μ*-*κ*^1^-N:*κ*^1^-N’ coordination mode of *s-trans* 1,4-diaminobutane (DAB) related ligands is characteristic to many coordination compounds including some platinum group metals [130,131]. The interesting feature found during synthesis of a heterobimetallic palladium–platinum complex with such bridging 4e donor *σ*,*σ*-N, N’ coordination fashion is the preservation of 1:1 Pd:Pt ratio in the crystal structure although due to the reported disorder in metal positions the authors did not preclude the coexistence of homo–dimeric Pd_2_ and Pt_2_ species in the same crystal structure (Figure 5a; FACKAV) [132].

An in situ synthesis of a butane-2,3-diiminato ligand via reductive coupling of acetonitrile at iridium in the presence of phosphine tridentate pincer type PC*sp*^2^P ligand (Figure 5b; SEYKOY) was reported by Burford et al. [133]. Except of *s-trans α*-diimine ligand the metallic center directly bonds to the carbon atom. Phosphine stabilized palladium complexes are recognized as active catalysts in asymmetric transformations, for example in a tandem cross-coupling C–H bond activation reactions [138,139,140]. However, nitrile reductive coupling at a late transition metal center is unusual.

Khan et al. reported the formation of stable diruthenium complexes of another example of non-innocent 1,2-diimine related ligand but with unusual synclinal and anticlinal conformations with torsions of 78.8(7) and 122.9(9)° along the N=C–C’=N’ fragment, respectively. These species do not show any oxidative cleavage. The clinal conformation resulted from the presence of four bulky substituents (i.e., two pendant phenyl and two pyridyn-2-yl groups; Figure 5c; LOMNIN) [134].

The *s-cis* conformation of 1,2-diimine in coordination compounds leads to the chelating mode. Zhai et al. studied copolymerization of ethylene with acrylate monomers with the use of functionalized 1,2-diimine. The reported structure of dipalladium complex shows the bidentate chelating 4e *κ*^2^-N,N’ binding mode of the diimine moiety (Figure 5d; ADOGEI) [135].

An example of a 1,2-diimine sterically blocked ligand is 1,2-diiminebenzene and its derivatives. The *s-cis* spatial arrangement of two conjugated imine groups is constrained this time by covalent bonding. Therefore, the ligand may form easily chelates. However, higher nuclearity can be afforded by additional bridging by the imine N atoms. In the reported structures the tridentate chelating bridging *μ*_2_-*κ*^2^:*κ*^1^ mode stabilizes diruthenium and tripalladium complexes (Figure 5e,f; KIGFUD and MICDAD) [136,137].

**Figure 6:**
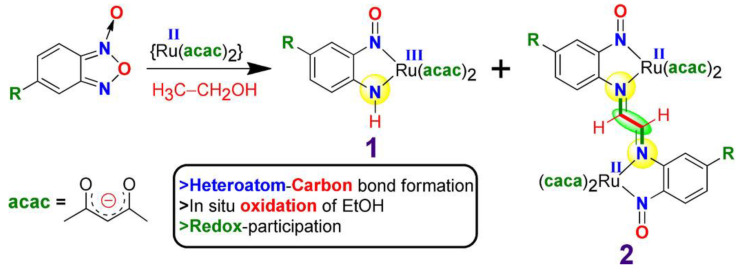
In situ solvent-mediated conversion of benzofuroxan affording 1,2-diimine non-innocent ligand [141]. Reprinted with permission from Ghosh, P.; Dey, S.; Panda, S.; Lahiri, G. K. Solvent-Mediated Functionalization of Benzofuroxan on Electron-Rich Ruthenium Complex Platform. *Chem. An Asian J.* **2018**, *13* (12), 1582–1593. Copyright (2018) John Wiley and Sons.

Ghosh et al. reported an interesting method of synthesis of 1,2 diimine ligands by via conversion of benzofuroxan at {Ru(acac)_2_}, which resulted in heteroatom–carbon formation via oxidation of alcoholic solvent, nucleophilic addition and intermolecular coupling (Figure 6) [141] (YIGHII and YIGHOO).

1,2-Diimine based ligands such as 1,4-diazabutadiene (DAB) have eight electrons prone to coordination. Two lone pairs of imine N atoms ligating in a *σ*-fashion and two pairs of *π* electrons on the -N=C–C=N- moiety. Therefore, DAB related molecules are frequently observed to form complexes with platinum group metals in a special way. The scheme of this bonding is as follows. The ligand entity acts as a chelating species to one metal center forming two *σ*-bonds, whereas the other metal atom is bonded via two *η*^2^-C=N bonds analogous to pentahapto (*η*^5^-) bonding mode (8e *μ*_2_-*κ*^2^-N,N’:*η*^2^-C=N:*η*^2^-C’=N’ mode; Scheme 1j–m). The planar five-membered chelate ring acts like a *η*^5^-cyclopentadienyl analogue.

**Figure 7:**
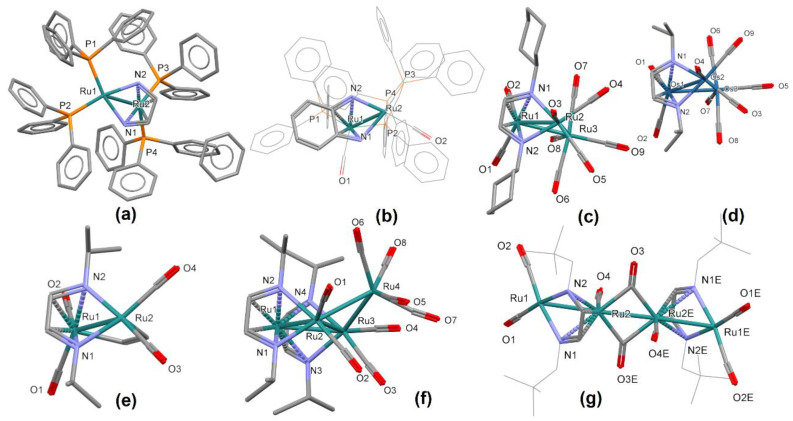
(**a**) The dual mode of coordination exhibited by 1,4-diazabutadiene ligands in binuclear complexes of with Ru. Chelating ligation and pentahapto-like (*η*^5^-) bonding [142]. (CSD Refcode FIMNAR); (**b**) cationic part of diruthenium complex with the *η*^5^ coordination mode [122] (CSD Refcode AROQAZ); (**c–f**) coordination mode 8e *μ*_2_-*κ*^2^-N,N’:*η*^2^-C=N:*η*^2^-C’=N’ of DAB ligands observed in metal carbonyl complexes: CSD Refcodes CUXFIK [143], GIRCAL [144], GLXRUA10 [145] and GLXRUB10 [146] and (**g**) unusual linear Ru_4_ cluster based on the 6e *μ*_2_-*κ*^2^-N,N’:*η*^2^-C’=N’ ligating 1,2-diimine ligand [143] (CSD Refcode DEXBIR). Solvent molecules and hydrogen atoms were omitted for clarity.

The examples of such coordination motif may be found both in simple 1,4-diazabutadiene ligands as reported by Abbel et al. (Figure 7a; FIMNAR) [142] as well as in the case of more extended ligands as in the 1,2-diiminobenzene Ru complex, which shows interesting electrochemical properties (Figure 7b; AROQAZ) [122].

Similar 8e donating *μ*_2_-*κ*^2^-N,N’:*η*^2^-C=N:*η*^2^-C’=N’ coordination scheme of DAB ligands are observed for example in oligonuclear metal carbonyl complexes (trinuclear Os [144] (GIRCAL), Ru [143] (CUXFIK), binuclear Ru [145] (GLXRUA10) and tetranuclear Ru [146] (GLXRUB10; Figure 7c–f)).

Keijsper et al. reported an unusual linear Ru_4_ cluster based on 6e *μ*_2_-*κ*^2^-N,N’:*η*^2^-C’=N’ ligating 1,2-diimine ligand and carbonyl bridges (Figure 7g; DEXBIR) [143].

**Figure 8:**
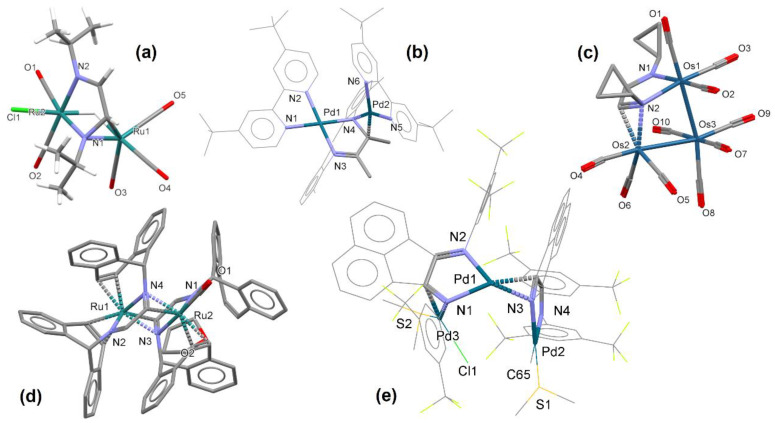
(**a**) Six electrons donor coordination mode (6e *μ*_2_-*κ*^2^-N,N’:*η*^2^-C’=N’) of the DAB ligand observed in the monohydride metal carbonyl complex of Ru [147] (CSD Refcode WAGKOE); (**b**) unusual three-membered PdCN ring with the *η*^2^-coordinated imidoyl group [148] (CSD Refcode OKAYUU); (**c**) triosmium 6e *μ*_2_-*κ*^2^-N,N’:*η*^2^-C’=N’ complex with the DAB related ligand [149] (CSD Refcode GIRDIU); (**d**) dimeric structure of the complex of the DAB ligand with ruthenium with the coordination mode (6e *μ*_2_-*κ*^2^-N,N’:*η*^2^-C’=N’) [150] (CSD Refcode YARYEY) and (**e**) mixed valence tripalladium 6e *μ*_2_-*κ*^2^-N,N’:*η*^2^-C’=N’ complex with the non-innocent acenaphthenequinone-based ligand [151] (CSD Refcode XOFZEY). Hydrogen atoms, solvent and counterion molecules were omitted for clarity.

However, the possible *η*^5^-like coordination mode may be disturbed, and “slipped” complexes are also observed with only one *η^2^*-C=N ligation and rarely encountered three-membered PdCN ring (Scheme 1l,m). An example may be a 6e donor *μ*_2_-*κ*^2^-N,N’:*η*^2^-C’=N’ coordination mode of DAB ligand, which was reported for monohydride metal carbonyl complexes of Ru [147] or described by Owen et al. dipalladium unit with the *η*^2^-coordination mode [148] (Figure 8a,b; WAGKOE, OKAYUU).

Zoet et al. described a series of ruthenium carbonyl coordination compounds with the DAB ligand with 4e, 6e and 8e donor coordination modes (Figure 8c; GIRDIU) [149].

The non-innocent DAB ligand was successfully applied in catalytic decarbonylation of aqueous formaldehyde with the production of hydrogen (Figure 8d; YARYEY) [150].

A slipped 6e *μ*_2_-*κ*^2^-N,N’:*η*^2^-C’=N’ bridging was reported by Zhou et al. (Figure 8e; XOFZEY) [151]. This trinuclear complex contained V-shaped palladium cluster stabilized by the bridging non-innocent acenaphthenequinone related ligand. The authors assumed a mixed valence for palladium centers because of the presence of a non-innocent ligand, which may be coordinated as monoanion radical or in the diamagnetic fully reduced dianionic form.

In contrast to 1,2-diimines (*α*-diketimines), the 1,3-diimines (N,N′-*β*-diketimines) are innocent ligands. However, this class of bidentate ligands can exist as a mixture of tautomers. They may coordinate in a chelating fashion forming six-membered chelate ring. 1,3-Diketimines are often referred to as HNacNac species. Bourget-Merle et al. reviewed the chemistry of *β*-diketiminato metal complexes [152]. The 1,3-diimine derivatives are frequently fused in macrocyclic compounds such as porphyrins or corrins. However, these groups of compounds are behind the scope of this review.

### 2.4. Coordination Chelating Pockets Made of Imine Group and Additional Ligating N/O/S Heteroatoms

The concept of joining the 1,2-diimine fragment with additional ligating N/O/S heteroatoms prone to coordination may lead to coordination chelating pockets (Scheme 2a–d).

**Figure 9:**
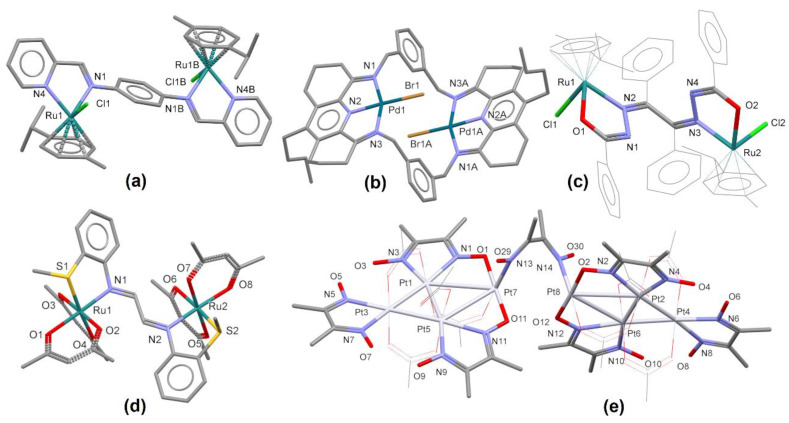
(**a**) Diruthenium complex with chelating *κ*^2^-N,N’ pockets [153] (CSD Refcode AZWQOL); (**b**) cyclic binuclear complex with robust pincer type 6e *κ*^3^-N,N’N” coordination mode [154] (CSD Refcode MOTZUT); (**c**) binuclear complex of ruthenium(II) arene benzil bis(benzoylhydrazone) with coordination chelating *κ*^2^-N,O pockets [155] (CSD Refcode QESSUF); (**d**) binuclear Ru complex with non-innocent diimine ligand binding through coordination chelating *κ*^2^-N,S pockets [123] (CSD Refcode KEHSOH); (**e**) octaplatinum complex with Pt_3_ metallic clusters stabilized by bridging and chelating dimethylglyoxime ligand molecules [156] (CSD Refcode MEMLUL). Hydrogen atoms, solvent and counterion molecules were omitted for clarity.

The combination with aromatic N atom may result for example in bidentate chelating 4e *κ*^2^-N,N’ mode as in diruthenium complex (AZEQOL) [153] or in a more extended pincer type species (6e *κ*^3^-N,N’N” mode, MOTZUT), which may be further used to construct macrocyclic oligonuclear ligands [154] (Figure 9a,b).

An example of imine fused with O ligating atom may be a series of acylhydrazone derivatives forming binucleating template for coordination of Ru(II) complexes, which were studied on antiproliferative activity and apoptosis induction showing higher cytotoxicity than *cis*-platin and low IC50 values against the screened cancer HeLa, MDA-MB-231 and Hep-G2 cell lines (Figure 9c; QESSUF) [155]. The presence of additional O atoms enabled formation of six-membered chelate rings.

An analogous concept was used by Chatterjee et al. who described an in situ formation of a binuclear Ru complex with bridging non-innocent ligand showing interesting electrochemical behavior caused by internal electron transfer (Figure 9d; KEHSOH) [123]. In this case an additional sulphur atom is introduced to give coordination chelating *κ*^2^-N,S pockets.

The modification with heteroatoms may also be applied at imine N atoms as in dimethylglyoxime, which has the 1,2-diimine fragment and additional oxygen atoms resulting in oxime tetradentate ligand. An octaplatinum complex of dimethylglyoxime was isolated as an intermediate state in the cluster core transformation for stabilization of Pt_3_ metallic clusters (Figure 9e; MEMLUL) [156]. However, this type of imine ligand modification together with various pincer type pockets is out of the scope of this review.

### 2.5. Oligonuclear Complexes Based on N_2_O_2_ Compartmental SB Ligands

#### 2.5.1. Classic bi- or Trinuclear Systems, which are N_2_O_2_-compartmental Schiff Bases

**Figure 10:**
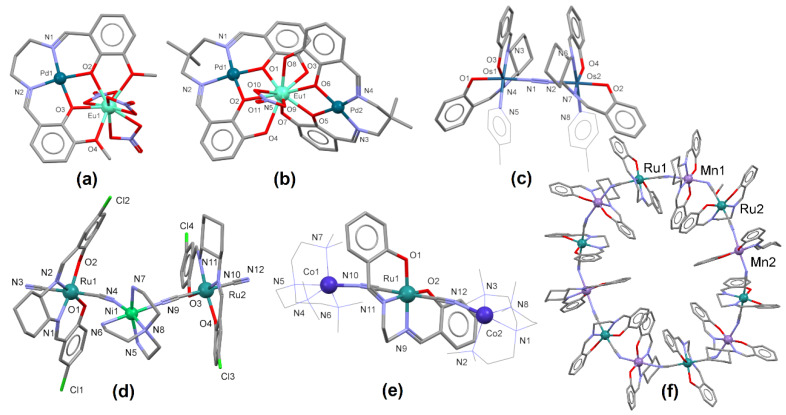
(**a**) Heterobinuclear Pd-Eu complex with bi-compartmental N_2_O_2_-O_2_O’_2_ Schiff base ligand [157] (CSD Refcode DEMPUJ); (**b**) heterotrinuclear Pd-Eu-Pd coordination compound with propeller like arrangement of two Schiff base ligands [44] (CSD Refcode DIPDAK); (**c**) diosmium *μ*-dinitrogen complex [158] (CSD Refcode MAHDAC); (**d**) heterobimetallic trinuclear cyano-bridged complex of bis(*μ*-cyanido)-dicyanido-bis(2,2’-[(*R*,*R*)-cyclohexane-1,2-diylbis(azanylylidenemethylylidene)]bis(4-chlorophenolato))-(tris(2-aminoethyl)amine)-nickel(II)-bi-ruthenium(III) [159] (CSD Refcode KILRIJ); (**e**) trinuclear Co-Ru-Co cyanido-bridged complex [160] (CSD Refcode BODHEJ); (**f**) dodecanuclear macrocyclic cyanido-bridged 3d-4d heterobimetallic complex [161] (CSD Refcode VIVCOV). Solvent molecules and hydrogen atoms were omitted for clarity.

The N_2_O_2_-compartmental Schiff base (Scheme 2e,f) are intensively used for studying of various transition metal complexes, however, there are only two structural reports on a series of crystal structures of classic heterobi- and heterotrinuclear systems in which the platinum group metals (Pt or Pd) were coordinated inside the N_2_O_2_-compartmental Schiff bases coordination site (Figure 10a,b; DEMPUJ and DIPDAK) [44,157].

These structures proved the flexibility of selected bi-compartmental N_2_O_2_-O_2_O’_2_ Schiff base skeletons to accommodate simultaneously the Pd(II) or Pt(II) ions (which demand a special planar tetragonal coordination environment) in the inner N_2_O_2_ pocket and lanthanide ions in the outer O_2_O’_2_ binding site. In case of heterotrinuclear Pd(II)-4f metal complexes the appropriate geometry for the auxiliary lanthanide ion was achieved by a propeller like arrangement of two Schiff base ligands resulting in linear trinuclear complexes that was previously observed for classic nonPGM homonuclear 3d or heteronuclear 3d-4f species enabling via bridges intermetallic interactions [162,163,164,165,166].

#### 2.5.2. Strategy of Addition of Auxiliary Bridging Ligands

To increase the nuclearity of mono- or binuclear units the strategy of addition of auxiliary bridging ligands was developed. In this approach the metal centers of mono-, bi- or oligonuclear complexes with N_2_O_2_ compartment Schiff bases may be bridged via various molecules or ions. For this application the following species were applied oxo, pyrazino, diphosphinidino and 1,1′-ferrocenediphosphiniminato [167] and even dinitrogen [158].

Man et al. studied the catalytic reduction of N_2_ and discovered that N⋯N coupling proceeds at room temperature in the presence of a SB ligand to provide a *μ*-dinitrogen complex (Figure 10c; MAHDAC) [158].

The auxiliary cyanide anions are frequently used in heteroleptic polymer systems bridging the metallic centers [168]. However, there are also known oligonuclear PGM species giving opportunity to intermetallic exchange interactions and resulting for example in single-molecule magnet properties [159,160,169,170,171].

Ru et al. reported the most recent examples of cyano-bridged heterobimetallic PGM compounds [159], in which the ferromagnetic coupling between metallic centers of Ru^III^ and Ni^II^ were enabled through the cyanide bridges (Figure 10d; KILRIJ).

The cyanide bridges were applied to study the phenomenon of switching off the single-molecule magnet upon complexation of mononuclear Co(II) coordination unit with salen Ru(III) system (Figure 10e; BODHEJ) [160].

#### 2.5.3. Nano-sized Magnetic Molecular Wheel

Cyanide anions bridging homo- or heterometallic systems may afford bi-, oligo- up to polymeric coordination structures [168]. However, Zhang et al. reported spectacular dodecanuclear macrocyclic cyanido-bridged 4d–3d heterobimetallic complexes. The complexes were synthesized via reaction performed at room temperature starting form precursors in the form of appropriate mononuclear Schiff-base complexes. It is an example of a chiral magnetic molecular wheel exhibiting the single molecular magnet (SMM) behavior (Figure 10f; VIVCOV) [161].

#### 2.5.4. Hybrid Materials Made of N_2_O_2_ Coordination Sites as Building Block

An interesting approach to oligonuclear complexes of fixed number of ligated metal centers is the application of fused ligands. The N_2_O_2_ compartment SB ligands exhibit excellent coordination properties. The combination of covalently bonded SB subunits by a “fusing agent” may give new ligands of higher number of coordination sites, both cyclic and acyclic (Scheme 2g–i). The geometry and coordination mode depend on the structure of the covalently bonded linker. Ligands fused by a stiff “fusing agent” frequently are flat, which favors intermolecular interactions in the form of stacking and intermetallic contacts providing additional tool in crystal engineering to stabilize the supramolecular structure. In the case of flexible “fusing agents” the molecule can change its conformation and adapt the shape to the guest molecules for example in molecular tweezers or helical structures. Finally, if the “covalent fusing” results in cyclic macromolecules, the conformation of the oligodentate ligand depends again on the shape adaptability of the molecular linkers giving opportunity to construct interlocked structures such as macrocycles, cages or molecular knots [172].

**Figure 11:**
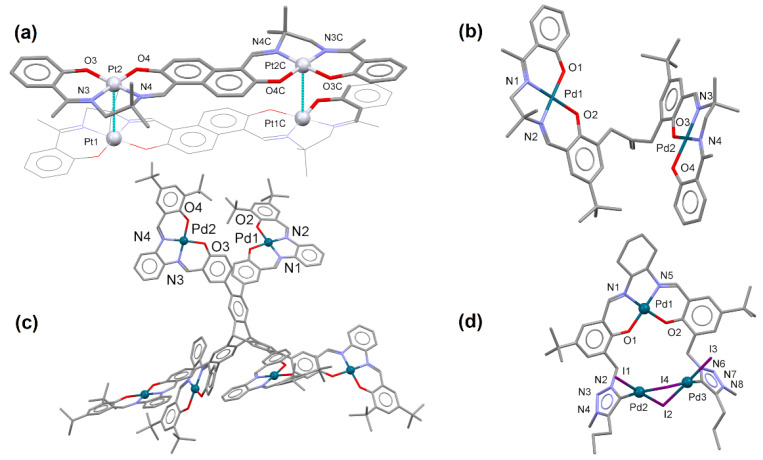
(**a**) Platinum(II) complex based on fused N_2_O_2_ Schiff base ligand and metallophilic interactions [41]. (CSD Refcode GEDBEZ); (**b**) binuclear coordination compound of palladium exhibiting mechanochromic photoluminescent properties [42] (CSD Refcode UMAWUC); (**c**) hexanuclear palladium triptycene based hexakis(metalsalphen) coordination compound [173] (CSD Refcode MIRSEO) and (**d**) trinuclear salen−mesoionic N-heterocyclic carbene hybrid complex of Pd(II) one of the symmetrically independent molecules [174] (CSD Refcode SELCIY). Solvent molecules and hydrogen atoms were omitted for clarity.

Houjou et al. presented a series of complexes of group 10 metal ions with ligands built of two N_2_O_2_ compartment SB subunits together with their luminescent properties [41]. The binuclear species interacted with each other via stacking and metallophilic interactions with the Pt–Pt distance of 3.434 Å (Figure 11a; GEDBEZ).

Simple heterobinuclear coordination compounds of Pd and Pt showed unusual mechanochromic photoluminescent properties making them attractive candidates as functional materials in memory devices (Figure 11b; UMAVUC) [42].

Reinhard et al. reported the synthesis and characterization of metal-assisted salphen organic framework (MaSOF) based on triptycenehexakissalicylaldehyde (Figure 11c; MIRSEO) [173]. To obtain microporous oligonuclear materials they used a template of triptycene moiety as a molecular skeleton base modified with the presence of N_2_O_2_ salphen type coordination pockets. The complexes were studied for the application as organic molecules of intrinsic microporosity (OMIM) materials. Such oligonuclear compounds have advantage over polymeric structures in application for gas sorption to be more soluble and defectless materials.

Schmid et al. applied the strategy of fusion of SB ligands with other type of functional materials for synthesis of oligonuclear salen−mesoionic N-heterocyclic carbene hybrids (Figure 11d; SELCIY) [174]. These compounds were studied on catalytic activity in the 1,4-addition of an oxindole to a nitroolefin.

### 2.6. Cyclic and Acyclic Macromolecules Made of Fused Schiff Base Units Resulting in Oligodentate Ligands

#### 2.6.1. Macrocycles

**Figure 12:**
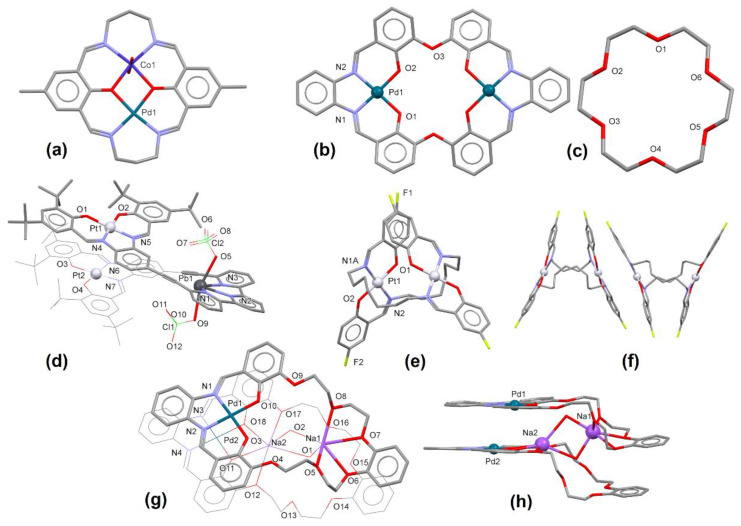
(**a**) A simple example of binuclear coordination compound based on calixsalen—Schiff base macrocycle [45] (CSD Refcode MIKQIJ); (**b**) 18-Crown-6-like binuclear metallohost based on two Pd metallosalen fragments [181] (CSD Refcode FARNIY); (**c**) macrocyclic 18-Crown-6 polyether [182] (CSD Refcode ABULOZ); (**d**) molecular tweezers based on Pt-salphen-terpyridine moiety for selective recognition of divalent cations helically closed on recognition of the Pb^2+^ ion [183] (CSD Refcode BORCES); (**e**) one of the symmetrically independent molecules and (**f**) side view at molecules of bis(*μ*-2,2’-{heptane-1,7-diylbis[azanylylidenemethanylylidene]}bis(4-fluorophenolato))-bi-platinum [184] (CSD Refcode YOLDEL) and (**g**) dimeric tetranuclear complexes of palladium(II) and alkali metal(I) ions based on crown ether-macrocycle with salophen related metallohost (**h**) and a side view [185] (CSD Refcode AYOYOC). Hydrogen atoms, solvent and counterion molecules were omitted for clarity.

Another approach is the synthesis of macrocyclic species [175]. The N_2_O_2_-based coordination pocket is an efficient building block for metallohost structures. For example, Robson-type macrocyclic complexes may be derived from condensation of achiral diamines and 2-hydroxy-5-methyl-benzene-1,3-dicarbaldehyde. Analogous to calixarene Li and Jablonski introduced a term of “calixsalen” in case of Schiff base macrocycles [176]. Many interesting ligand structures [177,178] and examples of PGM heterooligonuclear species (Figure 12a; MIKQIJ) were reported in the following papers [45,179,180]. In the case of PGM this approach for obtaining of homo- and heterooligonuclear species is particularly important, as these metals do not form oligonuclear complexes as easily as other metals.

Macrocyclic species may be extended into oligodentate metallohosts, which may be utilized for the recognition of alkali or alkaline earth metal ions such as crown compounds (Figure 12b,c; FARNIY and ABULOZ) [181,182].

The calixsalen molecules named analogous to calixarene were documented to self-assemble into larger species [186]. Akine and Nabeshima published an excellent review on cyclic and acyclic oligo(N_2_O_2_) ligands, however, they focused on the ligand’s structure and not on a particular group of metals [175].

#### 2.6.2. Metallohelical Structures

Doistau et al. reported on application of salphen related switchable molecular tweezers for selective recognition of divalent cations. The ligand is built of the terpyridine central part supplemented by two Pt-salphen pockets (Figure 12d; BORCES) [43,183]. A similar structure was synthesized based on the 4,5-dibromo-9,9-dimethyl-9H-xanthene precursor [187].

Cyclic binuclear clothespin shaped complexes of *trans*-bis(salicylaldiminato)platinum(II) and *trans*-bis(*β*-iminoaryloxy)palladium(II) were synthesized by Ikeshita et al. (Figure 12e,f; YOLDEL) [184] and Naito et al. [188,189].

Yoon et al. reported on interesting crown ether-macrocycle with incorporated building blocks of salophen moiety [185]. They succeeded in synthesis of dimeric tetranuclear complexes of palladium(II) and alkali metal(I) ions (Figure 12g,h; AYOYOC).

#### 2.6.3. Tweezers

**Figure 13:**
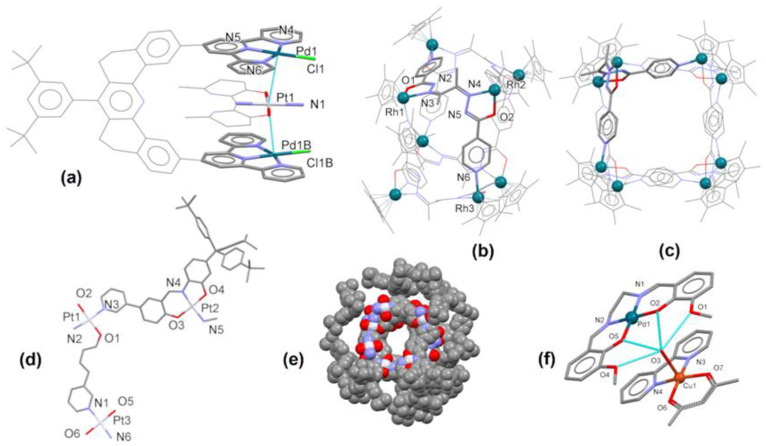
(**a**) Intermetallic contacts between Pd and Pt atoms in a host–guest adduct of a tweezer type dipalladium complex and a neutral platinum species [190] (CSD Refcode JAJWIB); (**b**) half sandwich M_8_(L)_4_ cubic cage of rhodium (L = 2,3-butanedione bis(isonicotinyl hydrazine)) (**c**) and a side view [191] (CSD Refcode UROREA); (**d**) one of oligonuclear Pt coordination units (**e**) and a view at nanotubular macrocycle aggregate presented as van der Waals spheres [192] (CSD Refcode BEHVUH) and (**f**) supercomplex built of square planar coordination entities acting as host receptors for aqua ligands coordinated by another coordination unit [193] (CSD Refcode TEGBUD). Hydrogen atoms, solvent and counterion molecules were omitted for clarity.

Supramolecular recognition is another approach, which may increase the nuclearity of complexes. A host-guest adduct of a tweezer type dipalladium and a neutral platinum species was synthesized. The structure is stabilized by stacking interactions and intermetallic contacts between Pd and Pt atoms (Figure 13a; JAJWIB) [190].

#### 2.6.4. Cages

Coordination cages are molecular self-assembly [194,195,196]. Liu et al. synthesized and characterized a series of half-sandwich polyhedral cages based on flexible SB ligands. For example 2,3-butanedione bis(isonicotinyl hydrazine) was used for synthesis of the octanuclear rhodium cubic cage (Figure 13b,c; UROREA) [191].

### 2.7. Cocrystals

Frischmann et al. presented a template-free approach for constructing nanotubular aggregates of macrocycles stabilized by stacking interactions [192]. They succeeded in the synthesis of molecular materials with substantial porosity based on discrete Pt_4_ macrocycle aggregates (Figure 13d,e; BEHVUH).

Bicompartment N_2_O_2_-O_2_O’_2_ SBs were an object for studying of supercomplexes and a construction of a second coordination sphere [193]. The square planar coordination entities act as host receptors for aqua ligands coordinated by another coordination unit (Figure 13f; TEGBUD). Such cocrystallization gives new opportunities in coordination chemistry.

### 2.8. Metallophilic Interactions in Oligonuclear Species

**Figure 14:**
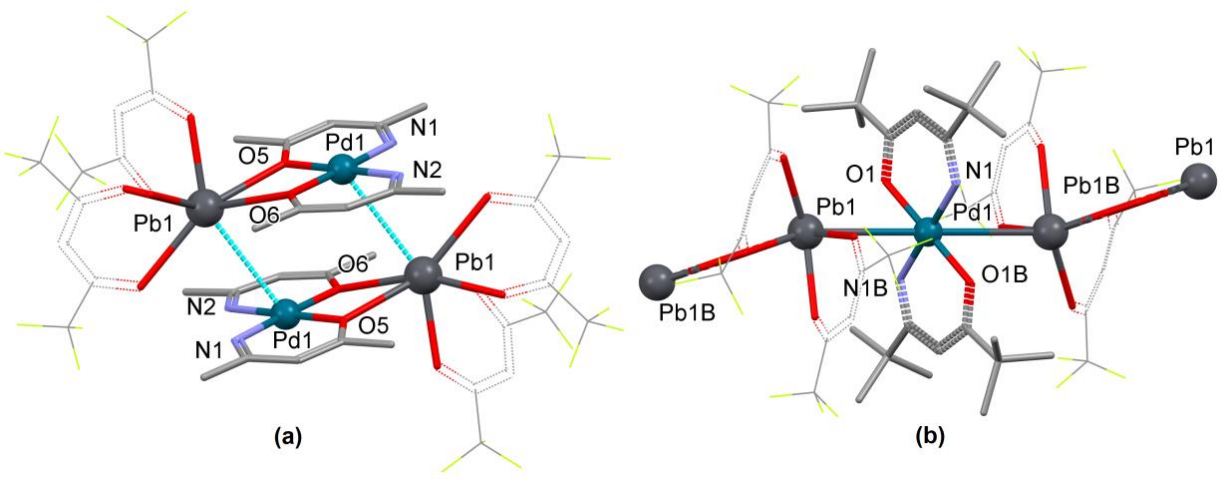
(**a**) Tetrameric (CSD Refcode HIJXEF) (**b**) and polymeric (CSD Refcode HIJWUU) structures based on metallophilic interactions with Pd–Pb distances of 3.551 and 3.459 Å, respectively [197].

Krisyuk et al. studied an interesting concept of utilization of metallophilic interactions to extend oligonuclear structures to higher order species up to coordination polymers basing on a series of Pd–Pb complexes. Dimers combined via metallophilic interactions formed tetramers or coordination polymers (Figure 14a,b; HIJXEF, HIJWUU). As the strength of metallophilic interactions is of order of hydrogen bonding’s, such new entities may result in interesting physicochemical properties [197].

## 3. Materials and Methods

### Methods of Database Analysis

The Cambridge Structural Database (CSD, version 5.41, update March 2020) [198] was used to extract the most recent achievements in the field of homo- and hetero- bi- and oligonuclear coordination compounds of platinum group metals (PGM) ligated by the imine group. The searches were carried out to retrieve complexes with at least two metallic centers in the coordination entity and with at least one metal belonging to PGM group, where PGM = Ru, Rh, Pd, Os, Ir or Pt coordinated by the imine group. The results were classified into distinct categories as presented in Scheme 1 and Scheme 2 and described in the manuscript. The database CSD Refcodes are given in the text of the manuscript next to the description of a particular crystal structure to provide reader with a quick reference. The author made an attempt to choose the most recent and most characteristic examples to present them to the reader. However, the author regrets not to include many other interesting studies due to a limited scope of this review. The molecular structures and schemes of coordination modes were visualized using Mercury 4.0 [199] and Chemsketch [200], respectively.

## 4. Conclusions

The aim of this review was to present the most significant concepts and strategies of construction and possible applications of the less studied but nevertheless important oligonuclear SB complexes of platinum group metals.

Oligonuclear imine platinum group metals (PGMs) complexes may be a promising platform for energy materials, especially in recently developing dye-sensitized solar cells (DSSCs) as well as in thermoelectric applications. The other field of emerging applications is gas storage materials based on organic molecules of intrinsic microporosity (OMIM) instead of polymers. Oligonuclear PGM complexes may find application as phosphorescent emitters in organic light-emitting diodes (OLEDs) because of existing metallophilic interactions in clusters. Oligonuclear complexes may also contribute to development of efficient optical or electrochemical sensors. The integration of Schiff base ligands and platinum group metals gives versatile possibilities of catalytic activity. Cyano-bridged structures may switch on/switch-off the single molecule magnet behavior. The oligonuclear entities have an advantage over the mononuclear or polynuclear species exhibiting usually better physicochemical properties than mononuclear ones and having higher solubility than polymers.

The future research directions in SB-PGM coordination chemistry in general should take into account the interplay between various weaker driving forces and additional more subtle interactions such as stacking or metallophilic interactions, which may result in novel useful oligonuclear materials.
